# Efficacy of Physiotherapy for Urinary Incontinence following Prostate Cancer Surgery

**DOI:** 10.1155/2014/785263

**Published:** 2014-04-24

**Authors:** Elżbieta Rajkowska-Labon, Stanisław Bakuła, Marek Kucharzewski, Zbigniew Śliwiński

**Affiliations:** ^1^University and Hospital Department of Rehabilitation, Institute of Physiotherapy at Gdańsk Medical University, Poland; ^2^Department of Descriptive and Topographic Anatomy, Medical University of Silesia, Ul. Jordana 19, 41-808 Zabrze, Poland; ^3^Head of Institute of Physiotherapy, Faculty of Health Sciences, Jan Kochanowski University at Kielce, Poland

## Abstract

The study enrolled 81 with urinary incontinence following radical prostate-only prostatectomy for prostatic carcinoma. The patients were divided into two groups. The patients in Group I were additionally subdivided into two subgroups with respect to the physiotherapeutic method used. The patients of subgroup IA received a rehabilitation program consisting of three parts. The patients of subgroup IB rehabilitation program consist of two parts. Group II, a control group, had reported for therapy for persistent urinary incontinence following radical prostatectomy but had not entered therapy for personal reasons. For estimating the level of incontinence, a 1-hour and 24-hour urinary pad tests, the miction diary, and incontinence questionnaire were used, and for recording the measurements of pelvic floor muscles tension, the sEMG (surface electromyography) was applied. The therapy duration depended on the level of incontinence and it continued for not longer than 12 months. Superior continence outcomes were obtained in Group I versus Group II and the difference was statistically significant. The odds ratio for regaining continence was greater in the rehabilitated Group I and smaller in the group II without the rehabilitation. A comparison of continence outcomes revealed a statistically significant difference between Subgroups IA versus IB. The physiotherapeutic procedures applied on patients with urine incontinence after prostatectomy, for most of them, proved to be an effective way of acting, which is supported by the obtained results.

## 1. Introduction

The International Continence Society defines urinary incontinence as involuntary leakage of urine that has been diagnosed objectively and is associated with additional hygienic and social problems [1]. The most common types of urinary incontinence following radical prostatectomy include stress incontinence, urge incontinence, and mixed incontinence. The European Association of Urology recommends two approaches to the management of these dysfunctions: noninvasive and invasive (surgical) [2]. Noninvasive modalities are considered first-line treatment during the first 6–12 months following prostatectomy [3]. Physiotherapy is administered especially early on after the onset of symptoms and in incontinence of moderate severity. At the same time, the approach to men with urinary incontinence following prostatectomy is not unified. An optimal rehabilitation plan requires prior determination of the type of urinary incontinence and of factors that may influence volitional control of micturition (motor control, musculo-fascio-skeletal relations, and behavioral factors) [4]. The choice of invasive versus noninvasive treatment depends on the severity and duration of symptoms and the type of urinary incontinence. Conservative modalities include physiotherapy techniques, the most common of which, used in male incontinence following prostatectomy, include pelvic floor muscle training (PFMT) with or without biofeedback (BFB), noninvasive pelvic floor electrical stimulation, extracorporeal magnetic innervation (ExMI), behavioral modification, and external penile compression devices [5–8]. A standardized program of pelvic floor muscle reeducation that would guarantee complete therapeutic success is yet to be developed. However, it is quite clear that a plan of pelvic floor muscle training should determine the level of exercise intensity with regard to exercise duration, the number and frequency of repetitions, and the type of load to induce permanent changes in the muscles [9–11]. Of no less importance for the final outcome of therapy are the patient's motivation, the correct execution of exercises, and compliance with the physiotherapist's orders [12].

The aim of the present study was to evaluate continence outcomes in a group of postprostatectomy males who underwent physiotherapy (Group I) as compared to a control group of males not undergoing physiotherapy (Group II) and to compare outcomes between Subgroup IA and IB, treated using different physiotherapy methods. Outcomes were also evaluated with regard to time between surgery and rehabilitation in Subgroup A (<3 months) and B (>3 months). The odds ratio for regaining continence in Group I versus (control) Group II was also evaluated.

## 2. Materials and Methods

The study enrolled 81 males aged 53–82 years (mean age 68 ± 6.65 years) with urinary incontinence following radical prostate-only prostatectomy for prostatic carcinoma.

The exclusion criteria included patients with an artificial pacemaker, musculoskeletal deformities making rehabilitation impossible, bleeding from the urinary bladder or the digestive tract, urinary tract infection, polyuria, uncontrolled diabetes mellitus, neurologic conditions affecting coordination and balance, lack of consent of the patient to commence therapy, and previous rehabilitation for urinary incontinence.

The patients were divided into two groups. Group I comprised *n* = 49 males aged 54–80 years (mean age 67.9 ± 6.81 years). The patients in Group I were additionally subdivided into two subgroups with respect to the physiotherapeutic method used.

Subgroup IA (*n* = 23) was made up of patients aged 54–77 (mean age 66.9 ± 7.07 years). These patients received a rehabilitation program consisting of three parts as follows:

- pelvic floor muscle training (PFMT) with biofeedback (PFMT + BFB) (once weekly for 20–30 min),
- pelvic floor muscle training according to spinal segmental stabilization principles (PFMT + SSS) (once weekly for 30 min),
- a set of exercises for the patient to perform on his own at home (3 times daily for 15–20 min).

The set of exercises comprised isolated contractions of PFMT and the urethral sphincter and particular pelvic floor muscle exercises according to the principles of spinal segmental stabilization training, performed in the lying, sitting, and standing positions.

The efficacy of PFMT with BFB was recorded graphically in a chart and numerically (in seconds and microvolts) using sEMG with a dual channel software-assisted Neuro Trac ETS device from Verity Medical Ltd. All patients from Subgroup IA had an individual anal probe mounted. A self-adherent reference electrode was positioned onto previously defatted akin at an electrically inactive site on the anterior superior iliac spine. The electrodes were FAD- and CE-certified.

Subgroup IB comprised a total of *n* = 26 patients aged 57–80 (mean age 68.8 ± 6.59 years) subjected to a rehabilitation program involving the following:

- PFMT without BFB according to the principles of segmental spinal stabilization training (twice weekly for 30 min),
- A program of home-based exercises identical to that for Subgroup IA.

Subgroup IB included those patients who had not agreed to have an anal probe mounted for exercises.

All patients followed a standardized and reproducible exercise program.

Therapeutic sessions in both subgroups were one-on-one meetings between patients and one physiotherapist at the Department of Rehabilitation, Gdańsk Medical University. Patients commuted for the sessions from their place of residence. The rehabilitation program was administered to patients who had developed urinary incontinence following a prostate-only prostatectomy and had been referred for rehabilitation by a urologist. The rehabilitation program in Subgroups IA and IB was divided into a number of stages.

Stage I, the same in both groups, began with educating the patient about the anatomy and physiology of the lower urinary tract and the respiratory system, the physiology of micturition, and muscle synergies between the musculoligamentous system of the pelvic complex and pelvic floor muscles.

In Stage II, also the same in both groups, the therapist carried out exercises with the patients according to the principles of segmental spinal stabilization training. Muscle activation involved kinesthetic awareness in tensing muscles while the patient maintained a neutral position of the spine. The exercises were performed at a slow pace, with a loading threshold of 20–25% of maximum MVC (maximum voluntary contraction strength), duration of muscle tensing of 5–10 seconds, and 7–10 repetitions. Training progression consisted of stimulating muscles of the external group at isolated positions while maintaining control of tensing, changes in starting positions for exercises, exercises in open and closed kinematic chains, sensorimotor exercises on unstable bases (mattress, theraband ball, and disks), and the addition of resistance and functional exercises. The duration of physiotherapy in both subgroups depended on the severity of incontinence. The therapy ended when continence was regained but did not last longer than one year.

The third stage was only implemented in Subgroup IA and involved the incorporation of biofeedback to monitor the parameters of pelvic floor muscle training.

The severity of incontinence was determined with the 1-hour and 24-hour pad test, where the number of pads used before and after therapy was recorded by patients in micturition diaries. Pelvic floor muscle tension was recorded and measured by sEMG (surface electromyography). The continence thresholds were 2 g in the 1-hour pad test and 4 g in the 24-hour pad test. Continence parameters were assessed at the beginning (baseline) and on completion of the therapy. Patients were divided into subgroups with regard to the amount of urine leaked to determine whether baseline urine loss would influence continence outcomes of the physiotherapy.

Group II, a control group, comprised 32 men aged 53–82 years (mean age 68.3 ± 6.49 years) who had reported for therapy for persistent urinary incontinence following radical prostatectomy but had not entered therapy for personal reasons. 1-hour and 24-hour pad tests were conducted when the patients reported for therapy and repeated at one year postsurgery. Information on continence outcomes was collected by phone on the basis of patients' self-reported subjective assessment.

Consent for conducting the study was given by the Independent Ethical Review Board for Scientific Research at Gdańsk Medical University (NKEBN 208/2011).

The study data were analyzed in STATISTICA 9.0. The Kolmogorov-Smirnov test was used to determine data distribution patterns. Quantitative characteristics were compared between the two populations with the Mann-Whitney *U* test. A statistical test for dependent samples was used to compare parameters for the same population (group) before and after treatment. The Chi² test was used to define correlations between two qualitative variables. A test for differences between two components of a structure served to verify the significance of particular frequency distributions (of patient numbers and percentages) of specific variable categories. Regression analysis was used additionally to assess odds ratios. A *P* value of *P* < 0.05 was regarded as statistically significant in all analyses.

## 3. Results

The 1-hour and 24-hour pad test were conducted in patients from Groups I and II before rehabilitation was started in order to test for homogeneity of the groups with regard to the amount of urine leaked. The tests did not reveal statistically significant differences between the groups (*P* = 0.329 for the 1-hour test and *P* = 0.105 for the 24-hour test).

**Figure 1:**
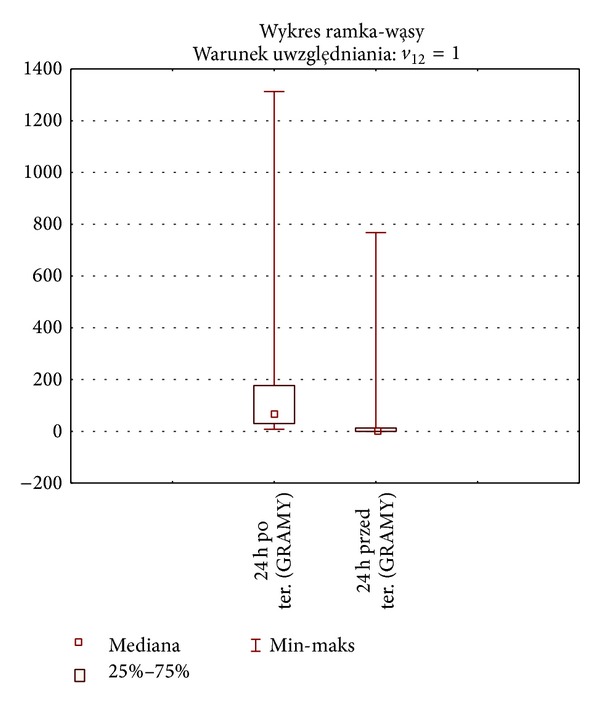
Differences in the amount of urine leaked before and after therapy for Group I (*n* = 49). Box-and-whiskers diagram. Case selection condition. 24-h test baseline (grams). 24 h test posttreatment (grams). Median. Min-max.

The 1-hour pad test was also used to provide an objective measure of the amount of urine leaked before and after rehabilitation in Group I patients (*n* = 49). The results showed a statistically significant difference (*P* < 0.001) in reducing the loss of urine. 24-hour pad test data also showed a statistically significant difference between measurements before versus after rehabilitation therapy (*P* < 0.001). The number of pads used per day before and after therapy (185 versus 58) was analyzed based on data from micturition diaries. A significant reduction in the number of pads used was observed following the therapy compared to baseline data (*P* < 0.001). Differences in demand for pads for the entire study group are presented in Figure 1.

sEMG traces obtained during biofeedback exercises for pelvic floor muscles were analysed statistically with regard to the following parameters: arithmetic mean of contraction voltages (*μ*V), mean deviation of contraction voltages (%), mean response time (s), arithmetic mean of relaxation voltages (*μ*V), mean deviation of relaxation voltages (%), and mean relaxation time (s). These indices were measured before and after therapy.

The only statistically significant (*P* = 0.03) difference among these values was in response times before and after treatment for continent versus incontinent patients. No significant differences were revealed with regard to the remaining indices.

**Figure 2:**
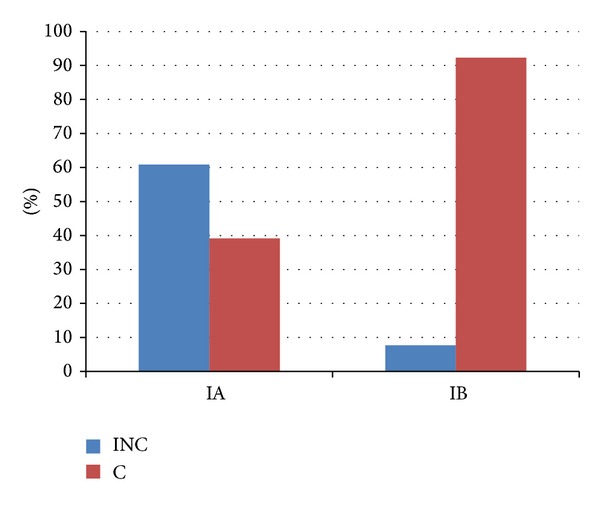
Comparison of outcomes of incontinence therapy (C versus INC) for Subgroup IA (PFMT + BFB) versus Subgroup IB (PFMT + SSS).

On completion of the therapy, continence (C) had been restored in 9/23 (39.1%) patients in Subgroup IA, while 14/23 (60.9%) patients remained incontinent (INC) at various degrees of severity. In Subgroup IB (*n* = 26), continence had been restored in 24/26 (92.3%) patients, and 2/26 (7.7%) were still not fully continent. These outcomes of different models of rehabilitation for urinary incontinence (PFMT + BFB versus PFMT + SSS) appear to be more favourable for Subgroup IB (Figure 2).

Analysis for the entire Group I shows a significant correlation between therapeutic outcome and the physiotherapeutic method used (Chi², *P* = 0.00007). There were considerably more incontinent patients in Subgroup IA (61%) than in Subgroup IB (8%) on completion of the therapy. The study did not demonstrate statistically significant differences with respect to incontinence between Subgroup IA versus IB (*P* = 0.08). A comparison of continence outcomes revealed a statistically significant difference between Subgroups IA versus IB (*P* = 0.007). The highest percentage of continent patients following physiotherapy was in Subgroup IB, at 24/26 (92%).

We also studied the effect of time between prostatectomy surgery and commencement of physiotherapy on continence outcomes. The patients were accordingly divided into two subgroups. Subgroup A comprised patients who began rehabilitation within 3 months of surgery and Subgroup B included those who reported for rehabilitation three months or more after surgery. The patients were additionally classified as continent (C) and incontinent (INC). The analysis showed that the best continence outcomes were obtained in patients who entered rehabilitation within three months of surgery. Differences in the odds ratio for continence between Subgroup A (<3 months) versus B (>3 months) were in favor of those who entered rehabilitation earlier. The odds ratio for the recovery of continence in Subgroup A amounted to 4.88 while in the group B 0.29. These results emphasize that the chances for regaining full continence decrease considerably with increasing time between prostatectomy surgery and entering rehabilitation.

Analysis of data for the entire study population (*n* = 81) revealed differences in continence/incontinence between males from Group I versus Group II (33/49 versus 4/32; 89% versus 11%). Superior continence outcomes were obtained in Group I versus Group II (33 versus 4) and the difference was statistically significant (*P* = 0.0001). The odds ratio for regaining continence was greater in rehabilitated Group I and smaller in the group II without the rehabilitation. The odds ratio for the recovery of continence in Group I amounted to 2.06 while in the Group II 0.15.

**Figure 3:**
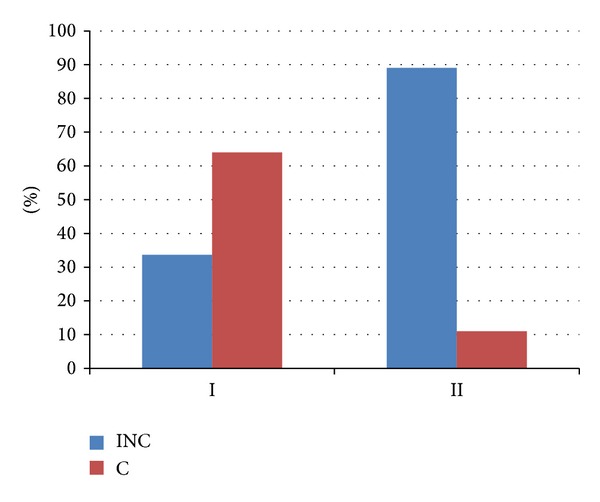
Therapy outcomes (C versus INC) in Group I versus Group II.

The results are presented in Figures 1, 2, and 3. 1-hour and 24-hour pad testing did not reveal significant differences in the amount of urine loss between Group I (rehabilitated) versus Group II (not rehabilitated) before rehabilitation began. The consumption of pads decreased significantly (*P* < 0.001) on completion of the therapy compared to baseline. On completion of the therapy, 9/23 patients in Subgroup IA were continent compared to 24/26 in Subgroup IB. BFB produced statistically significant changes (*P* = 0.03) only in response time as measured by sEMG. Incontinence following therapy was still present in 14/23 patients in Subgroup IA and 2/26 patients in Subgroup IB. Superior continence outcomes were noted in patients who had entered rehabilitation within 3 months of prostatectomy surgery compared to those who had taken longer to begin rehabilitation (Subgroup A versus Subgroup B; OR 4.88 versus 0.29). Analysis of the entire study population (*n* = 91) revealed superior continence outcomes in Group I versus Group II (33/4), representing better odds for regaining continence in rehabilitated patients versus nonrehabilitated controls (OR 2.06 versus 0.15).

## 4. Discussion

The management algorithm for urinary incontinence in men following radical prostatectomy is not fully specified with regard to both the choice of physiotherapy methods, timing, and duration of therapy and exercise intensity [13–20]. The risk of incontinence may be influenced not only by the surgical method and operative technique but also by other attendant factors [18, 21], such as age, prostate volume, tumor stage, history of TURP (transurethral resection of the prostate), and volume leaked following catheter removal [22–24]. Porru et al. described a much higher rate of resolution of incontinence and dribbling symptoms in a rehabilitated group versus a control group [25]. The authors recommend physiotherapy exercises on account of their efficacy and ease of execution [25]. Guidelines regarding the timing of physiotherapy vary between authors. The timing of therapy is important for continence outcomes [17, 23]. In our study, patients reported for physiotherapy at various times following prostatectomy surgery. They were admitted for rehabilitation on the basis of a referral note for physiotherapy from their urologists. The data show that Subgroup A (<3 months between surgery and rehabilitation) had greater odds for regaining continence than Subgroup B (>3 months). Physiotherapy for continence was more beneficial in patients who started treatment up to 3 months following prostatectomy versus those who entered rehabilitation after a longer interval (Subgroup A versus Subgroup B, OR 4.88 versus 0.29). The chances for complete recovery of continence decreased considerably with increasing interval between surgery and beginning of rehabilitation. A review of the literature showed that both the duration of supervised therapy and session frequency are not uniform across published papers [7, 14, 16, 22, 26, 27]. In the present study, as in van Kampen et al.'s, the therapy continued until micturition control had been regained and also did not exceed 12 months [22]. All patients from the group undergoing rehabilitation had one-on-one sessions with the same physiotherapist. Similar to other studies, two sessions a week were held and each lasted on average 30 minutes [3, 23]. Patients were instructed to repeat a set of exercises at home 3 times a day in sessions not exceeding 20 min. Overgård et al. demonstrated that superior increments in muscle diameter and strength could be obtained with twice-daily versus once-daily exercise sessions [7]. The 1-hour and 24-hour pad tests were used to compare urine loss between Group I and II at baseline (before rehabilitation commenced), but did not reveal statistically significant differences (*P* = 0.329 for 1-hour pad test; *P* = 0.105 for 24-hour pad test), which means that the two groups were comparable with respect to the amount of urine leaked. van Kampen et al. observed that low urine loss immediately following catheter removal may be prognostic for rapid recovery of continence following prostatectomy [22]. Published studies have used the 1-hour pad test to assess the amount of urine leaked. This tool has been recommended for that purpose by ICS [28, 29]. Börgermann et al. point out that a variety of criteria are used to classify a postprostatectomy patient as “dry” [30]. For some researchers, a patient is continent when he does not use any protection while for others a continent patient does not use one and/or more pads [19, 30]. The present study relied on micturition diaries to obtain data on pad consumption in Group I (*n* = 49). Pad consumption decreased after therapy in a statistically significant manner compared to baseline (*P* < 0.001). Micturition diaries are another effective and noninvasive tool for assessing incontinence. Researchers vary in their opinions about the optimal number of records. Some recommend keeping a diary for 7 days [31], while others make do with 4-day records [32]. In the present study, patients kept micturition diaries for 4 days. Methods of treatment of iatrogenic urinary incontinence following prostatectomy in men include biofeedback [16, 17]. However, this is not a method in itself. It is only a tool helping patients achieve exercise goals. The sound and vision make it easier to monitor exercise tasks. Our study used sEMG to measure and monitor pelvic floor muscle contractions. Various outcomes have been reported with this kind of therapy. Ribeiro et al. used biofeedback combined with sEMG monitoring of muscle activity to train pelvic floor muscles. The biofeedback group registered much better continence outcomes compared to the control group [19]. Some reports have stated no effect of BF on the efficacy of continence recovery [13, 33]. In our study, PFM training combined with biofeedback was also used in Subgroup I. Performance of exercises was monitored by the registration of action potentials from muscles during contraction and relaxation. Of all indices monitored, a statistically significant difference (*P* = 0.03) was only observed with regard to response time at baseline versus on completion of therapy, which may indicate improved timing in neuromuscular coordination. However, the additional incorporation of biofeedback in Subgroup I did not increase the efficacy of continence recovery, similar to the findings of other studies [13, 33]. Some authors believe that patients with incontinence following prostatectomy may regain continence spontaneously within one or even two years following the surgery [22, 30, 34]. In order to ensure an objective assessment of our therapy, we followed up both rehabilitated and nonrehabilitated patients. More patients regained continence in the physiotherapy group, with 33/49 (67.3%) continent patients in Group I, compared to 16/49 (32.7%) incontinent patients. In Group II (nonrehabilitated control group), continence was confirmed in 4/32 (12.5%) patients and 28/32 (87.5%) remained incontinent. In our opinion, these comparative data encourage the use of physiotherapy in men with this dysfunction. Studies of incontinence present results of single-modality therapies [14, 17, 20, 35, 36]. The present study compared continence outcomes in patients treated with a combination of two methods. Patients in Subgroup I entered a biofeedback-enhanced exercise program for pelvic floor muscles based on spinal segmental stabilization training, while those in Subgroup II only practiced pelvic floor muscle exercises based on segmental spinal stabilization training. The addition of biofeedback in Subgroup I did not bring about a dramatic increase in the efficacy of continence recovery.

Pelvic floor muscles are morphologically classified as skeletal muscles and so they adapt to exertion in the same way as do other muscles from this group. Regular training leads to an increase in muscle bulk, which can also be observed in pelvic floor muscles. Endurance and strength training for incontinent patients aims to change muscle morphology by increasing their diameter, improve neurological indices by increasing the numbers of active motor neurons and the number of excitatory stimuli, and improve muscle tone [11, 37, 38]. Factors that slow down muscle responses to exertion include age, which is also associated with slower response times. Older (>70) men were found to have grossly limited ability for migration of the urethra against the symphysis pubis and had blurred anatomical borders of the urethral sphincter system [39]. Increased diameter and endurance of pelvic floor muscles are achieved using the principles of training designed for other skeletal muscles as pelvic floor muscles are made up of striated tissue. It is not completely understood how these training principles translate into changes in the continence muscles. Good neuromuscular coordination appears to be of immense importance for normal pelvic floor muscle function. The muscles of the pelvic floor have different points of origin and attachment in that region. Some attach directly to bones and others to fasciae and ligaments. In describing the functions of pelvic floor muscles, Shafik points out that the external anal sphincter (EAS), external urethral sphincter (EUS), and the bulbospongiosus muscle (BC) have their origins on the puborectalis muscle (PR) and act as a mutually dependent muscle complex. His study demonstrated that pelvic floor muscles behave like one muscle, which does not preclude the possibility of them working independently from one another [40]. A number of strategies for activating pelvic floor muscles are available. These strategies are based on isolated muscle tensing, functional training and motor control, the use of muscle synergies involving the transversus abdominis muscle, and the use of appropriate breathing patterns [41]. Some researchers believe that the pelvic floor muscles are part of a structural complex that forms a lumbopelvic cylinder comprised of the pelvic floor, respiratory diaphragm, transversus abdominis muscle, and lower spine, including the multifidus muscle [42]. Mutual coordinated relationships between these components result in increased intraabdominal pressure, which has a stabilizing role. A rise in intraabdominal pressure precedes an increase in intraurethral pressure [43]. A relationship between PFM activity and intraabdominal pressure contributing to urethral elevation was demonstrated by Junginger et al. [44]. To prevent urine leakage, the actively contracting pelvic floor muscles increase urethral closing pressures and are accordingly viewed not only as stabilizers but also as promoters of abstinence. Other researchers emphasize the importance of the transversus abdominis (TrA) as a significant structure taken advantage of in exercises for incontinent patients. The function of the transversus abdominis in generating pressures transmitted onto the pelvic floor muscles has been extensively studied. The hypothesis that the roles depend on the site of origin/attachment of a muscle is yet to be fully confirmed [45]. Junginger et al. demonstrated that elevation of the bladder neck is consistently seen when pelvic floor muscles are tensed in combination with mild tensing of TrA. Inhibition of urethral elevation was seen when abdominal muscles were slightly tensed in combination with a slight Valsalva maneuver and raising of the head [44]. The authors suggested that their findings should be incorporated into plans for the rehabilitation of patients with urinary incontinence. They indicated that PFM and TrA were jointly activated only during submaximal tensing, while 100% tensing caused an excessive rise in intraabdominal pressure as a result of the activation of all abdominal muscles, which lowered the bladder neck. Thus, the authors concluded that only submaximal PF tensing should be achieved therapeutically [44]. Sapsford suggested that a contraction of the transversus abdominis may facilitate tensing the pubococcygeal part of the levator ani [46].

Other researchers suggest, on the basis of their findings, that tensing abdominal muscles facilitates the tensing of PF muscles. Jones believes that viewing the role of pelvic floor muscles in a broader perspective paves the way for other rehabilitation modalities that can now be used for this purpose [42]. She notes that physiotherapy methods used for the selection of exercises for incontinent patients and those employed in the treatment of back pain and spinal instability have a lot in common as both take advantage of connections between muscle groups in the lumbar region, abdominal muscles, and pelvic floor muscles.

The role of this muscle complex is to produce and control tensions in response to changes in intraabdominal pressure. Richardsoan et al. point out that good control of pelvic floor muscle tension is an important element of the restoration of abdominal stabilization in incontinent patients [47]. In their work with patients, physiotherapists do not “train” only one muscle. Approaching the dysfunction as a local problem manifesting only at one site in the body is not sufficient in view of the current knowledge of physiology, biomechanics, and neurology. One has to agree with Dr. Lee, who notes that “understanding musculoskeletal disorders requires an awareness of how loads are transferred by the body and how a disturbance in one part may affect the functioning of the entire system” [48]. Many authors have tried, first theoretically and subsequently in practice, to explain the mechanisms underlying mutual effects that muscular and fascial structures exert on one another. Researchers who have worked to unravel the integrity of these structures include F. Meziera and K. Tittel. They have presented certain connections related to muscle chains whose mutual relations stemmed from functional links [49]. A similar view was presented by Myers in “Anatomy Trains,” where he pointed to musculofascial connections as the bearers of mutual links. Kassolik and Andrzejewski similarly sought links in musculofascial connections in their tensegration massage theory [50]. All these authors based their proposals on the assumptions of tensegration formulated by Buckminster Fuller. With this approach, the human body, particularly the musculoskeletal system, is viewed as a whole subject to continuous shifts between stability and mobility, which is associated with constantly shifting tensions and the emergence of compressions in the human body both in movement and at rest. These aspects are explained by D. Ingber [51]. Approaching the musculoskeletal system as a spatial musculonervoskeletal structure is conducive to finding muscle connections that can be used in working with patients and whose role can be understood solely by reference to their structural and functional relations. Similar assumptions were presented by D. Lee based on earlier work by Vleeming. D. Lee introduced an easily understood division of muscle trains into an external and internal group. Connections between muscle trains as presented by her are used in restoring stability of the lumbar spine and pelvic girdle, but have also been employed in the treatment of urinary incontinence [48]. The conception of stabilization and appropriate choice of exercises has been presented by other authors, including Dr. Richardsoan et al. [47]. Urinary incontinence is associated, among others, with functional weakening of the pelvic floor muscles, which are regarded as deep (central) stabilization structures. Jones emphasizes that, when the identification of pelvic floor muscles is disturbed, the use of other muscles forming the central stabilization cylinder results in tensions being automatically transferred onto the muscles of this region [42]. The pelvic and sacral bone are the sites of attachments of approximately 35 muscles; therefore, their mutual relations play a role in the movement and stabilization of this region of the body as well as other segments. Our approach to working with incontinent males following prostatectomy is based on principles used in central stabilization training, especially with regard to training motor functions, proposed for that purpose by Richardsoan et al. Their training principle involves a system of three successive stages of rehabilitation offering progressively increasing loads and always taking into account local segmental control. In line with Richardson's recommendation, tensions used during training sessions with patients did not exceed 30% of the MVC. Movements were performed at a slow pace in order to activate tonic muscles [42, 48, 52]. The duration of sustained tensing was 10 s with 5–10 repetitions, in accordance with Libenson's instructions for this type of training [52]. The rehabilitation also involved “functional respiratory patterns” as proposed by Sapsford for strengthening pelvic floor muscles in incontinent women [46]. The author refers to mutual relations in the inspiratory and expiratory phase between the abdominal muscles, respiratory diaphragm, and pelvic floor muscles. Exercises during the respiratory phase were also used during sessions with patients by Ribeiro et al. [19]. Published strategies of management of urinary incontinence have been changing, from local approaches to the problem [53–57] towards concepts linking pelvic floor muscles with other muscle groups forming a functional whole [46–48]. State-of-the-art knowledge affords a better understanding of relationships within the musculofascioligamentous system in the space of the pelvic floor and the cylinder formed by the respiratory diaphragm, abdominal muscles, and lower back. Imbalance between these structures produces functional changes. The ability to grasp mutual relationships in the locomotor system enables for the therapeutic use of the influence of particular elements on other components that are often not directly related.

## 5. Conclusion

The findings of the present study show that a physiotherapy program can improve or fully restore continence. Data for the entire Group I suggest that early institution of physiotherapy after a prostatectomy procedure contributed to regaining continence. Continence outcomes were better in the rehabilitated group compared to nonrehabilitated controls. The study tools (pad testing, micturition diaries, and sEMG) proved useful for our analyses and presentation of the results of the study.
